# Defect-Induced Photoluminescence Blinking of Single Epitaxial InGaAs Quantum Dots

**DOI:** 10.1038/srep08898

**Published:** 2015-03-10

**Authors:** Fengrui Hu, Zengle Cao, Chunfeng Zhang, Xiaoyong Wang, Min Xiao

**Affiliations:** 1 National Laboratory of Solid State Microstructures and School of Physics, Nanjing University, Nanjing 210093, China; 2 Department of Physics, University of Arkansas, Fayetteville, AR 72701, USA

## Abstract

Here we report two types of defect-induced photoluminescence (PL) blinking behaviors observed in single epitaxial InGaAs quantum dots (QDs). In the first type of PL blinking, the “off” period is caused by the trapping of hot electrons from the higher-lying excited state (absorption state) to the defect site so that its PL rise lifetime is shorter than that of the “on” period. For the “off” period in the second type of PL blinking, the electrons relax from the first excited state (emission state) into the defect site, leading to a shortened PL decay lifetime compared to that of the “on” period. This defect-induced exciton quenching in epitaxial QDs, previously demonstrated also in colloidal nanocrystals, confirms that these two important semiconductor nanostructures could share the same PL blinking mechanism.

Photoluminescence (PL) blinking has long been observed in a variety of single optical emitters^1^,^2^ ranging from nanocrystal (NC), quantum dot (QD), dye, polymer, porous silicon to fluorescent protein, and most recently even to carbon nanotube^3^, nitrogen-vacancy center^4^ and silicon carbide^5^. This intriguing optical phenomenon was first reported in single colloidal CdSe NCs in 1996^6^ and soon explained by a theoretical model^7^ proposing that the blinking “off” period should originate from nonradiative Auger recombination of a charged exciton. Guided by this pioneering model and its modified forms^1^,^8^, partially or completely nonblinking CdSe NCs have been realized by either adding molecular species in solution to remove the carrier traps responsible for charging^9^,^10^ or imposing alloyed compositions in synthesis to suppress the Auger recombination effect^11^. Another major progress in PL blinking control was made in “giant” CdSe/CdS NCs, where the reduced overlap of the carrier wave functions and the alloyed core/shell interface due to the adoption of a thick CdS shell effectively suppressed the Auger recombination of charged excitons^12^,^13^,^14^. This has significantly elevated the PL quantum yield of the blinking “off” period from a charged exciton, which is normally associated with a shorter PL decay lifetime than that of the “on” period from a neutral exciton^15^,^16^. More interestingly, a new kind of PL blinking “off” period was additionally discovered with an equal PL decay lifetime to that of the “on” period, which was attributed to nonradiative quenching of a neutral exciton from the NC absorption state to the defect site^16^. So far, this defect-related model has been widely discussed in the recent PL blinking studies of a variety of semiconductor NCs^17^,^18^,^19^,^20^ and it is important to see whether such behavior can be extended universally, or at least partially, to any other optical emitter systems.

The Auger recombination effect can be intrinsically ignored in epitaxially-grown semiconductor QDs due to their smooth potential-energy functions for the quantum confinement of photo-excited carriers^11^,^21^, as signified by the highly-efficient charged-exciton^22^ and biexciton^23^ emissions routinely measured on a single QD level. As a consequence, PL blinking was rarely observed in single epitaxial QDs, but it could be triggered by introducing physical defects on the sample surface, with the PL “off” periods lasting for hundreds of milliseconds and longer^24^,^25^,^26^,^27^,^28^,^29^. PL blinking process at time scales ranging from tens of nanoseconds to tens of milliseconds was also discovered in single epitaxial QDs from the second-order photon correlation measurements^30^,^31^. Meanwhile, it was recently reported that the PL linewidth of single epitaxial QDs could be broadened from several to tens and hundreds of μeV due to the spectral diffusion effect^32^,^33^. Both the short-time scale PL blinking and the spectral diffusion effects were tentatively explained by carrier fluctuations among limited numbers of trapping sites located either in the vicinity of the QDs or at the capping layer/blocking barrier interface^31^,^32^,^33^.

Here, we report two types of PL blinking behaviors observed in single epitaxial InGaAs/GaAs QDs around defect sites intentionally created in the sample structure. For the first type of PL blinking, the “on” and “off” periods have the same PL decay lifetimes, resembling what was previously observed in “giant” colloidal CdSe/CdS NCs. The “off” period can be attributed to the trapping of hot electrons from the GaAs absorption state to the defect site, which is further supported by its shorter PL rise lifetime relative to that of the “on” period. For the “off” period in the second type of PL blinking, the electrons relax from the QD emission state into the defect site, leading to an equal PL rise lifetime and a shortened PL decay lifetime compared to those of the “on” period.

## Results

### Sample structure and basic PL blinking properties

**Figure 1:**
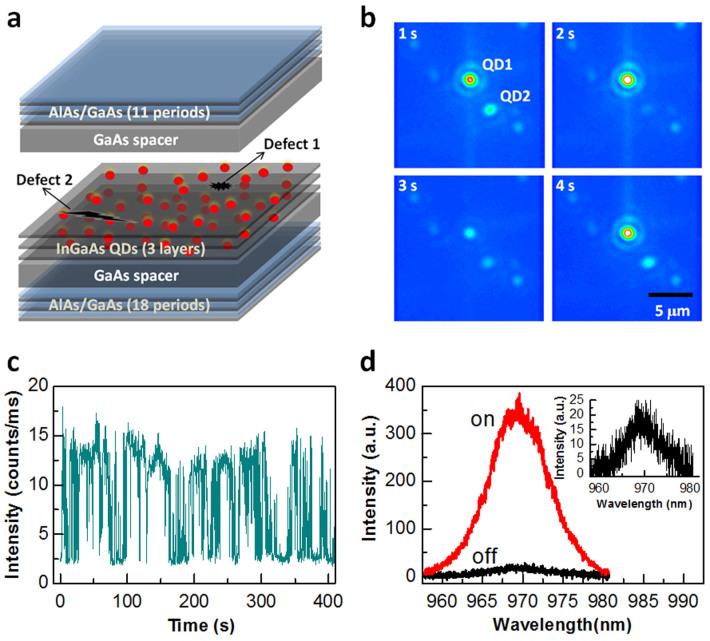
Sample structure and basic PL blinking properties. (a), Schematic of the sample structure with three layers of InGaAs QDs embedded between two planar mirrors. “Defect 1” and “Defect 2” denote two kinds of defect sites created in the GaAs barrier and the InGaAs QD, respectively. (b), PL images taken successfully from 1–4 s for several InGaAs QDs with blinking (“QD1” and “QD2”) and nonblinking (all the other QDs) PL behaviors. (c), PL intensity *versus* time trace measured for “QD1” with a binning time of 100 ms. (d), PL spectra measured for the blinking “on” and “off” periods of “QD1”. A magnified PL spectrum of the blinking “off” period is shown in the inset.

The InGaAs QDs studied here were epitaxially grown between two planar mirrors to efficiently collect their PL signals (see Fig. 1a and Methods). As reported previously^29^, PL blinking could be triggered in a single InGaAs QD by intentionally making scratches on the sample surface. In Fig. 1b, we present four PL images taken successively from 1–4 s at the temperature of ~4 K for several single InGaAs QDs excited at ~800 nm by a picosecond pulsed laser. It can be seen over the measurement time that the two single QDs denoted by “QD1” and “QD2” demonstrate a pronounced PL blinking behavior, while the PL signals from all the other single QDs are relatively stable. In Fig. 1c, we plot the PL intensity *versus* time trace measured for “QD1”, where the random switching of its PL intensity between the “on” and “off” periods can be clearly resolved (see Supplementary Fig. S1 for similar time traces of four more QDs). The blinking “off” periods of “QD1” and all the other single QDs studied in our experiment are still associated with a dim PL intensity level, similar to the one commonly observed in “giant” CdSe/CdS NCs^12^,^13^,^16^. This can be further confirmed in Fig. 1d from the two PL spectra measured for the “on” and “off” periods of “QD1” (see the inset for a magnified PL spectrum of the “off” period).

**Figure 2:**
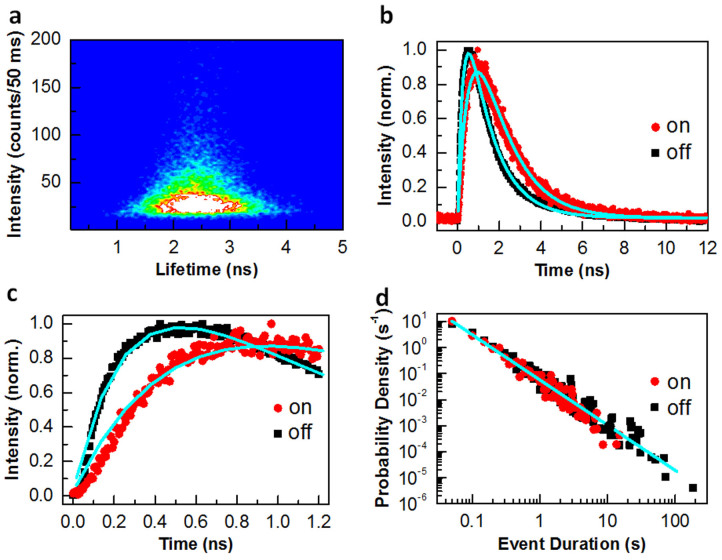
First type of PL blinking behavior. (a), FLID image of a single InGaAs QD with a vertical alignment of the lifetime-intensity data points. The PL intensity and average lifetime were calculated for a binning time of 50 ms. (b), Transient PL curves measured for the blinking “on” and “off” periods. Each of these two curves is fitted by the function, 
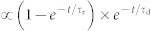
. (c), Similar transient PL curves to those shown in b but plotted within a shorter time window. (d), Probability densities of the “on” and “off” times both fitted by the same power-law function, ∝*t*^−α^.

**Figure 3:**
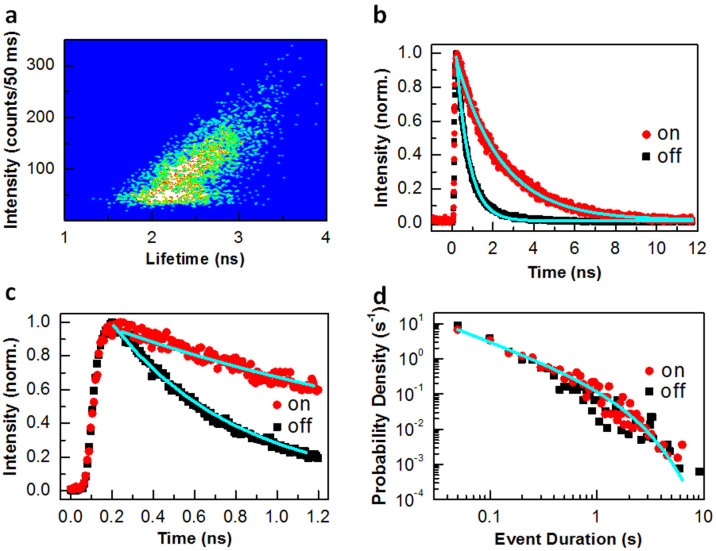
Second type of PL blinking behavior. (a), FLID image of a single InGaAs QD with a positively-sloped alignment of the lifetime-intensity data points. The PL intensity and average lifetime were calculated for a binning time of 50 ms. (b), Transient PL curves measured for the blinking “on” and “off” periods. The decay part for each of these two curves is fitted by a single-exponential function, 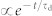. (c), Similar transient PL curves to those shown in b but plotted within a shorter time window. (d), Probability densities of the “on” and “off” times both fitted by the same truncated power-law function, 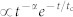.

For a sample size of 3 mm × 3 mm with several scratches on the surface, we were able to discover >50 blinking QDs, 18 of which had good signal-to-noise ratios in their PL intensity *versus* time traces for a reliable data analysis. The PL blinking behaviors of these 18 QDs can be classified into two categories according to the pattern of their fluorescence lifetime-intensity distributions (FLIDs). For 8 of the 18 blinking QDs studied, the lifetime-intensity data points are vertically aligned, as can be seen in Fig. 2a from the FLID image of a representative QD (see supplementary Fig. S1(a) for its PL intensity *versus* time trace). This first type of PL blinking behavior features the same PL lifetimes of the “on” and “off” periods despite their dramatically different PL intensities. For the second type of PL blinking behavior observed in the rest 10 of the 18 blinking QDs studied, the lifetime-intensity data points are distributed along a positively-sloped line, as can be seen in Fig. 3a from the FLID image of a representative QD (see supplementary Fig. S1(b) for its PL intensity *versus* time trace). This positive correlation implies that the “on” period is associated with a longer PL lifetime than that of the “off” period.

### First type of PL blinking behavior

**Figure 4:**
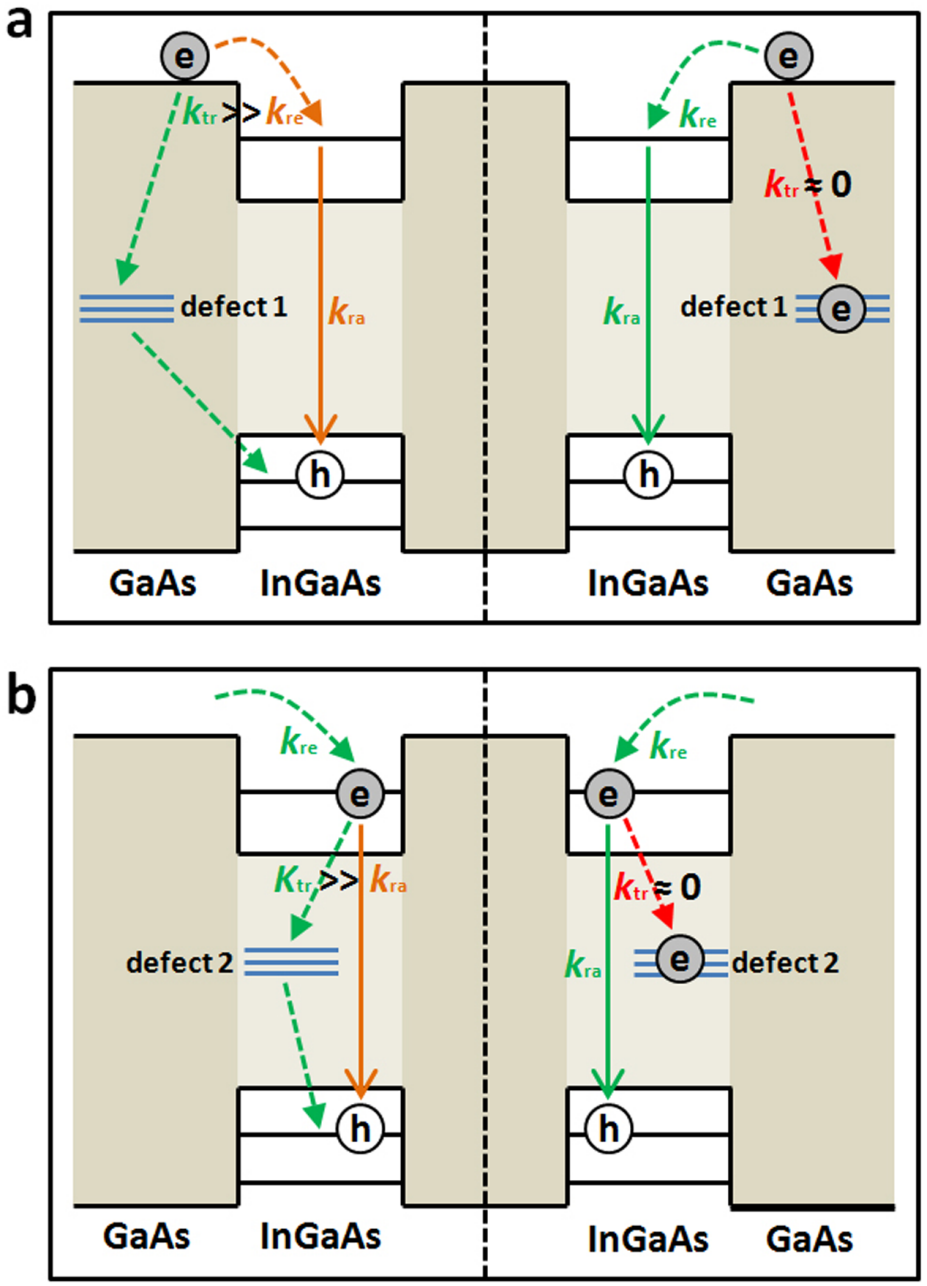
Defect-induced PL blinking model. (a), For the first type of PL blinking, the “off” period (left panel) is caused by the trapping of hot electrons from the GaAs barrier into the defect site. The “on” period (right panel) appears when the defect site is filled by an unpaired electron. (b), For the second type of PL blinking, the “off” period (left panel) is caused by the electron trapping from the QD emission state into the defect site. The “on” period (right panel) appears when the defect site is filled by an unpaired electron.

The first type of PL blinking behavior shown in Fig. 2a was previously observed only in the “giant” CdSe/CdS^16^ and the type-II InP/CdS^20^ NCs, where the “off” period was attributed to the trapping of hot electrons from their absorption state to a defect site. This scenario can be also applied to our case when assuming that a defect site is located in the surrounding GaAs barrier of a single InGaAs QD (corresponding to “defect 1” in Fig. 1a). As shown in the left panel of Fig. 4a, the hot electrons in GaAs could be captured by an empty defect site with a rate *k*_tr_ that is much larger than *k*_re_ of their relaxation rate into the QD emission state. Under this condition of 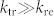, the chance for an exciton to be formed in the QD is greatly reduced, so that its PL signal would be dwelling at the weakly-emitting “off” period. The trapped electron in the defect site could recombine with the remaining hole in the QD nonradiatively before the next photo-excitation event and the “off” period would be extended continuously. It could happen that, after an electron-hole pair is created in the GaAs, only the electron flows into the QD area to fill the defect site, as shown in the right panel of Fig. 4a. Now with a trapping rate of *k*_tr_ ≈ 0, any hot electron excited subsequently would relax into the emission state and recombine radiatively with the hole to trigger the PL blinking “on” period. The trapped electron could leave the defect site once it encounters an extra hole either in the GaAs or the QD due to its unbalanced capture of photo-excited carriers^34^ and thus, the transition between the “off” and “on” periods would be recycled.

The schematic model shown in Fig. 4a naturally predicts that the blinking “on” and “off” periods should have a similar PL decay rate of *k*_ra_ and different PL buildup rates of *k*_tr_ + *k*_re_. For the “off” period with 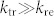, the rate for the hot electrons to be captured by the emission state is mainly determined by *k*_tr_. For the “on” period with *k*_tr_ ≈ 0, the hot electrons relax into the emission state with a rate of *k*_re_. In Fig. 2b, we plot two transient PL curves measured for the blinking “on” and “off” periods, each of which is fitted by a function form, 
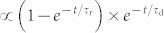
, with *τ*_r_ and *τ*_d_ being the rise and decay lifetimes, respectively^35^. The PL decay lifetime of ~1.42 ns fitted for the “on” period is close to that of ~1.26 ns for the “off” period. On the other hand, the PL rise lifetime of the “on” period is ~1.23 ns, which is significantly longer than the ~0.34 ns value of the “off” period. These apparently different PL rise lifetimes between the “on” and “off” periods are further emphasized in Fig. 2c, where we have plotted the transient PL curves within a shorter time window than that in Fig. 2b.

For simplicity, we only place one energy level (emission state) in Fig. 4a for the electrons in a single QD although there should exist several higher-lying excited states. In principle, the relaxation time (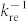) of hot electrons from the GaAs to the emission state of a single QD should be close to that from its higher energy levels, which is normally on the time scale of sub-hundred picoseconds^36^,^37^. Then the elongated rise lifetime of ~1.23 ns for the transient PL of the “on” period in Fig. 2b and c implies that a trapped electron in the GaAs defect site may strongly delay the hot-electron relaxation into the emission state possibly due to the Coulomb repulsion effect^15^,^38^.

### Second type of PL blinking behavior

For the “giant” CdSe/CdS NCs studied before, the defect sites were proposed to interact with only the absorption states^16^, which can be easily explained by their locations in the CdS shell responsible for the absorption of excitation photons. For the epitaxial InGaAs QDs studied here, the defect sites can be created by the physical scratches either in the GaAs barrier, or in the InGaAs QDs (corresponding to “defect 2” in Fig. 1a) to trigger the second type of PL blinking behavior (Fig. 3a). As shown in Fig. 4b, the hot electrons now relax from the GaAs barrier into the QD emission state with a similar rate of *k*_re_ for the “off” (left panel) and “on” (right panel) periods. For the “on” period with *k*_tr_ ≈ 0, the emission-state electron would recombine with the hole at a radiative rate of *k*_ra_. For the “off” period with 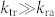, the nonradiative trapping rate of *k*_tr_ would be dominant for the exciton recombination, resulting in a weakly-emitting PL signal from the QD. In Fig. 3b, we plot two transient PL curves measured for the blinking “on” and “off” periods, respectively, and a higher-resolution look of these two curves is presented in Fig. 3c within a shorter time window. It can be clearly seen that the two rise parts cannot be distinguished from each other, with a lifetime value determined mainly by the detector time resolution. The decay parts of these two transient PL curves are each fitted with a single-exponential function (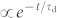), with the PL decay lifetimes of *τ*_d_ being ~2.20 ns and ~0.62 ns for the “on” and “off” periods, respectively.

### PL blinking statistics

For the InGaAs QD with the first type of PL blinking behavior in Fig. 2a, the probability densities for its “on” and “off” times in Fig. 2d can be fitted by the same power-law function (∝*t*^−α^) with *α* = 1.73. For the InGaAs QD with the second type of PL blinking behavior in Fig. 3a, the probability densities for its “on” and “off” times in Fig. 3d can be fitted by the same truncated power-law function 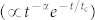 with *α* = 1.14 and *t_c_* = 1.45 s. Similar to Fig. 2 and Fig. 3, we present in Supplementary Fig. S2 and Fig. S3 the optical properties of another two InGaAs QDs with the first and second types of PL blinking behaviors, respectively. For the QD in Fig. S2, the probability densities of its “on” and “off” times can both be described by the truncated power-law functions. For the QD in Fig. S3, the probability density for its “on” times follows a power-law distribution, in contrast to that of its “off” times with a truncated power-law distribution. We can conclude that, although the blinking statistics have been utilized successfully in “giant” CdSe/CdS NCs to differentiate between the defect- and Auger-induced exciton quenching pathways^16^, they are not applicable in the epitaxial InGaAs QDs studied here to tell whether the electrons are trapped from the GaAs absorption state or the QD emission state into the defect site.

## Discussion

The emission peaks of all the PL dots in Fig. 1b are within the wavelength range of ~940–960 nm, where there exist transverse modes in the microcavity structure and the number of observable dots is greatly reduced^29^. Moreover, only one in a thousand PL dots possessed the blinking behavior^29^, which makes us believe that each blinking dot should correspond to a single InGaAs QD. However, since there are three layers of InGaAs QDs separated by 17 nm in this sample structure, we cannot completely rule out the possibility that each blinking dot might be a localized cluster of several QDs, especially when their emission peaks are all within the ~940–960 nm wavelength range. For each of the two types of PL blinking behaviors, there were PL dots with either binary (most likely from a single QD) or continuous distribution of the time-dependent PL intensity. Even if the “continuous” dots were indeed from QD clusters, the trend extracted from their “on”- and “off”-period PL dynamics was just the same as that from the “binary” dots, so that the defect-induced PL blinking model established in Fig. 4 would not be significantly affected.

As reported previously in other epitaxial QDs with the PL blinking behavior^24^,^25^,^27^,^28^, the PL spectra shown in Fig. 1d are relatively broad with a full width at half maximum of ~13 meV, in contrast to the tens of μeV value measured for nonblinking QDs in the same sample^36^. As shown in Supplementary Fig. S4, the “on”-period PL intensity of a representative blinking QD increases almost linearly with the increasing laser power density from ~10–100 W/cm^2^. Since we mainly used a laser power density of ~50 W/cm^2^ in our experiment, the chance for the biexciton generation in a single QD would be very small. On the other hand, the energy separation between a neutral and a charged exciton was measured to be ~3–4 meV from single nonblinking InGaAs QDs in a similar sample. So it is possible that, in addition to neutral exciton, the charged exciton can also contribute to the broad PL spectra observed here in our blinking QDs due to the unbalanced capture of photo-excited carriers^34^. For single epitaxial QDs without obvious PL blinking behavior, the PL linewidth could be only broadened to <100 μeV due to the spectral diffusion effect caused by charge fluctuations in a very limited number of nearby undetectable defects^32^,^33^. In contrast, the drastic PL blinking behavior of single epitaxial QDs was always associated with a PL linewidth of tens of meV^24^,^25^,^27^,^28^,^29^, which might reflect the strong interaction between the QDs and a large number of surrounding defect sites that were sometimes visually detectable^25^,^29^.

We assume in Fig. 4 that at most one electron could be trapped in a defect site and, in reality, it may take more electrons to completely fill the surrounding defect sites. Random fluctuations in the extra electric field posed by these trapped electrons^25^, especially during their trapping into and de-trapping from the defect site, could cause significant spectral diffusions of both neutral and charged excitons to explain the relatively broad PL spectrum shown in Fig. 1d for the single InGaAs QDs studied here and other single epitaxial QDs reported previously with the PL blinking behavior^24^,^25^,^27^,^28^. This explanation is consistent with the theoretical calculations and experimental measurements performed on single epitaxial InP/GaInP QD where the presence of extra charges in the surrounding wetting layer caused a significant broadening of its PL spectrum even at 3 K^39^. It should also be the number variation of these trapped electrons that gives rise to a continuous distribution of the lifetime-intensity data points in the FLID images of Fig. 2a and Fig. 3a. Consequently, a binary description employed here with the “on” and “off” periods is only a convenient choice that is not strictly appropriate. It could be imagined that, with the increasing laser power, the filling probability for the defect site would be increased to favor the appearance of more “on” periods, as demonstrated in the Supplementary Fig. S5 and also observed in the previous PL blinking studies of epitaxial QDs^24^,^26^,^29^.

It should be noted that for the current sample studied in our experiment, the only way to create blinking QDs is to intentionally make physical scratches. There also exist some other techniques to create blinking QDs in a more controllably way, such as by adding impurities, applying electric fields and using thin capping layers^25^,^31^,^32^. The physical scratches created here on the sample surface might not only introduce point and line defects, but also partially relax the strain in the wetting layer to change the QD environment. This large family of defects cannot be just modeled with the midgap states that interact with either the GaAs absorption state or the QD emitting state. For example, the PL signal could be completely quenched if the QD structure is severely damaged by the physical defects. Moreover, both the electron absorption and emission states associated with a single QD could be connected to the defect sites, which may occur in a majority of the blinking InGaAs QDs studied in our experiment with extremely low “on”-period PL intensities.

To summarize, we have induced PL blinking behavior in single InGaAs QDs by intentionally making physical scratches on the sample surface. The PL linewidth of tens of meV possessed by these blinking QDs implies that they can no longer be treated as quantum-confined artificial atoms, but their PL blinking properties have provided us with a deeper understanding on the interaction processes between the QD charge carriers and the surrounding environment. We have observed two types of PL blinking behaviors in the QDs due to nonradiative trapping of photo-excited electrons from the GaAs absorption state and the QD emission state into the defect sites, respectively. The first type of PL blinking, featuring an equal PL decay lifetime of the “on” and “off” periods, was observed previously also in the “giant” CdSe/CdS^16^ and the type-II InP/CdS^20^ NCs. In contrast, the “on” period in the second type of PL blinking has a larger PL decay lifetime than that of the “off” period, which is a missing behavior not possessed by the “giant” CdSe/CdS and the type-II InP/CdS NCs with a defect-related picture. However, this positive correlation between the PL intensity and the PL lifetime was encountered occasionally in previous PL blinking studies of traditional CdSe NCs, where the emission-state excitons were proposed to interact with fluctuating nonradiative decay channels^40^,^41^. We believe that nonradiative trapping of photo-excited carriers by the defect sites should be a universal PL blinking mechanism in the two important semiconductor nanostructures of colloidal NCs and epitaxial QDs. Similar connections may be extended to some other optical emitters, such as nitrogen-vacancy center^4^ and silicon carbide^5^, whose exact origins of the weakly-emitting “off” periods in a PL blinking process are still elusive at this time.

## Methods

### Sample fabrication

A detailed procedure of fabricating the InGaAs QDs confined in a planar microcavity with the molecular beam epitaxy technique was reported previously^29^. In brief, 18 periods of AlAs(770 Å)/GaAs(644 Å) were grown on a 5000 Å GaAs buffer layer to build the bottom mirror. After the growth of another GaAs layer of 5583 Å, the following structure of In_0.35_Ga_0.65_As(30 Å)/GaAs(170 Å)/In_0.35_Ga_0.65_As(30 Å)/GaAs(170 Å)/In_0.35_Ga_0.65_As(30 Å)/GaAs (6228 Å) was deposited to form three layers of InGaAs QDs. Finally, 11 periods of AlAs(770 Å)/GaAs(644 Å) were grown as the top mirror.

### Optical measurements

The sample was mounted in a He flow cryostat operated at ~4 K and the 800 nm output of a 76 MHz, picosecond Ti:Sapphire laser was focused on the sample surface at an incident angle of ~45°. The laser power density was set at ~50 W/cm^2^ to make sure that the QD PL was far from the saturation regime, as verified by the linear increase of the PL intensity and the unchanged PL dynamics even after this power density had been increased by three times. The sample PL was collected vertically by a 60× microscope objective and sent through a long-pass (>920 nm) optical filter to a 0.5 m spectrometer. A charge-coupled-device camera was equipped after the spectrometer for the PL imaging and spectral measurements, and an avalanche photo diode was alternatively used for the transient PL measurements employing a time-correlated single-photon counting (TCSPC) system with a time resolution of ~300 ps. The TCSPC system was operated under the TTTR mode so that the arrival times of each photon relative to the laboratory time and the laser pulse time could be both obtained, which allowed us to plot the PL intensity *versus* time traces and the transient PL curves, respectively.

## Author Contributions

X.W., C.Z. and M.X. conceived and designed the experiments. F.H. and Z.C. performed the optical experiments. F.H. and X.W. analyzed the data. X.W., F.H. and M.X. co-wrote the manuscript.

## Supplementary Material

Supplementary Information Supplementary Info
