# Supplementary material for: Defect-Induced Photoluminescence Blinking of Single Epitaxial InGaAs Quantum Dots
Source: Sci Rep. 2015 Mar 10;5:8898. doi: 10.1038/srep08898 (PMC4354043; doi:10.1038/srep08898)
Supplement: Supplementary Information — Supplementary Info [file srep08898-s1.doc]

**Supplementary Information**

Defect-Induced Photoluminescence Blinking of Single Epitaxial InGaAs Quantum Dots

Fengrui Hu^1^, Zengle Cao^1^, Chunfeng Zhang^1^, Xiaoyong Wang^1*^, and Min Xiao^1,2*^

*^1^National Laboratory of Solid State Microstructures and School of Physics, Nanjing University, Nanjing 210093, China*

*^2^Department of Physics, University of Arkansas, Fayetteville, AR 72701, USA*

*e-mail: wxiaoyong@nju.edu.cn, mxiao@uark.edu


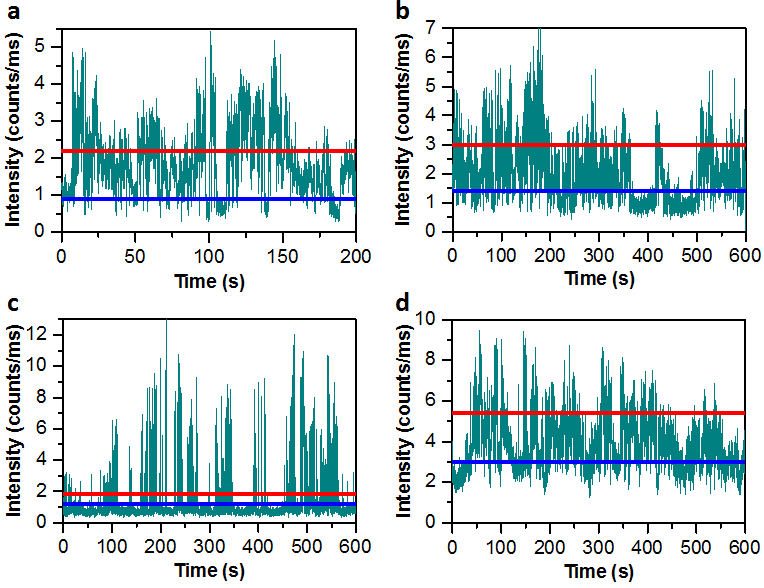


**Supplementary Figure S1.** PL intensity *versus* time traces of four single InGaAs QDs. The four curves shown in (a), (b), (c) and (d) were measured for the QDs whose other PL blinking properties are shown in Fig. 2, Fig. 3, Fig. S2 and Fig. S3, respectively. The red (blue) line in each figure marks the intensity threshold above (below) which the data points are classified into the PL blinking “on” (“off”) periods.


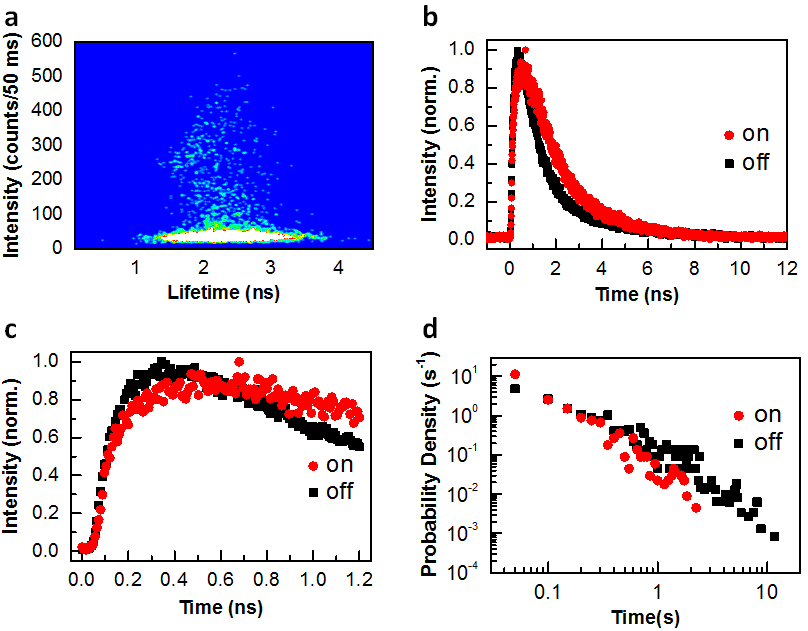


**Supplementary Figure S2.** The first type of PL blinking behavior measured for a single InGaAs QD whose PL blinking *versus* time trace is shown in Fig. S1(c). (a) FLID image with a vertical alignment of the lifetime-intensity data points. The PL intensity and average lifetime are calculated for a binning time of 50 ms. (b) Transient PL curves measured for the blinking “on” and “off” periods. (c) Similar transient PL curves to those shown in (b) but plotted within a shorter time window. (d) Probability densities of the blinking “on” and “off” times.


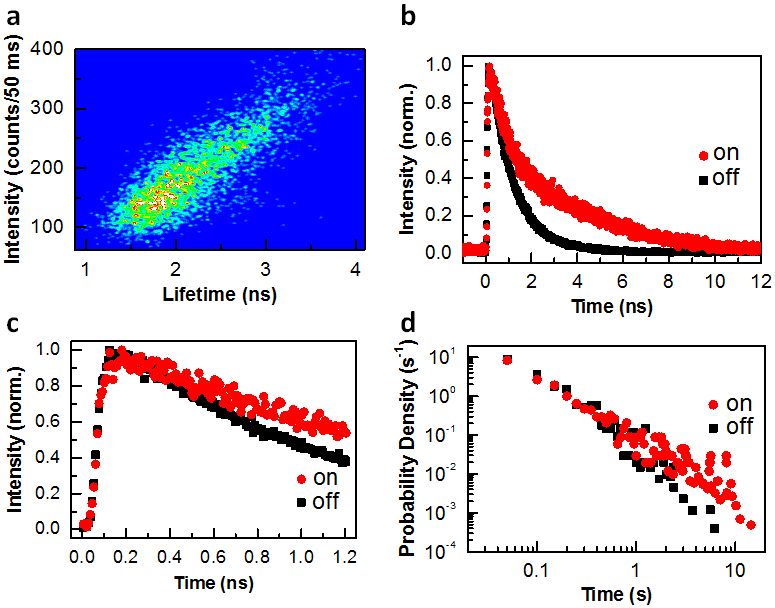


**Supplementary Figure S3.** The second type of PL blinking behavior measured for a single InGaAs QD whose PL blinking *versus* time trace is shown in Fig. S1(d). (a) FLID image with a positively-sloped alignment of the lifetime-intensity data points. The PL intensity and average lifetime are calculated for a binning time of 50 ms. (b) Transient PL curves measured for the blinking “on” and “off” periods. (c) Similar transient PL curves to those shown in (b) but plotted within a shorter time window. (d) Probability densities of the blinking “on” and “off” times.


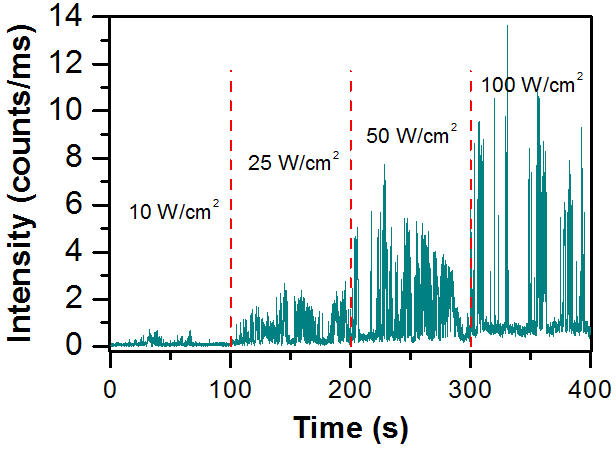


**Supplementary Figure S4.** PL intensity *versus* time trace of a representative blinking QD excited with increasing laser power densities.


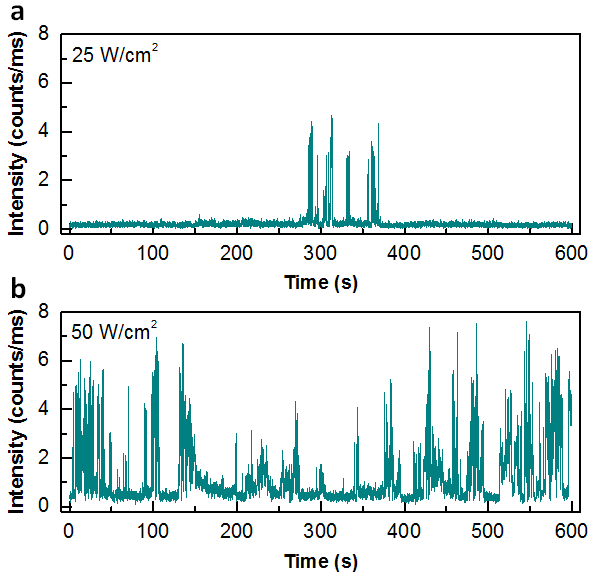


**Supplementary Figure S5.** PL intensity *versus* time traces of a single InGaAs QD excited at the power densities of (a) 25 W/cm^2^ and (b) 50 W/cm^2^, respectively.
